# Mobile Dental Delivery System: An Effective Protocol for Hygiene and Disinfection

**DOI:** 10.3390/ijerph17051603

**Published:** 2020-03-02

**Authors:** Damien Offner, Gabriel Fernandez De Grado, Marion Strub, Laure Belotti, Stéphanie Deboscker, Anne-Marie Musset

**Affiliations:** 1 Université de Strasbourg, Faculté de Chirurgie Dentaire, 8 rue Ste Elisabeth, F-67000 Strasbourg, France; 2 INSERM (French National Institute of Health and Medical Research), UMR 1260, Regenerative Nanomedicine (RNM), FMTS, F-67000 Strasbourg, France; 3 Pôle de Médecine et Chirurgie Bucco-Dentaires, Hôpitaux Universitaires de Strasbourg (HUS), 1 Place de l’Hôpital, F-67000 Strasbourg, France; 4 Service d’Hygiène Hospitalière, Laboratoire d’hygiène, Hôpitaux Universitaires de Strasbourg (HUS), 1 Place de l’Hôpital, F-67000 Strasbourg, France; 5 Service d’Hygiène Hospitalière, Equipe opérationnelle d’hygiène, Hôpitaux Universitaires de Strasbourg (HUS), 1 Place de l’Hôpital, F-67000 Strasbourg, France

**Keywords:** anesthesia, general, dental equipment, water microbiology, dentistry, operative rooms, pediatric dentistry, vulnerable populations

## Abstract

Mobile dental delivery systems (MDDSs) are receiving growing interest for reaching isolated patients, as well as in dental care for fragile and hospitalized patients, with the advantage of being able to be used from room to room or during general anesthesia (GA) in an operating room. Therefore, ensuring the care safety is crucial. The aim of this study was to elaborate and assess an MDDS maintenance protocol, containing the management of dental unit waterlines and adapted to specific conditions such as dental care under GA. A step-by-step protocol was established and implemented for an MDDS used during dental care under GA in children. Samples of the output water were collected at J0, J+1, 3, 6, 12, and 24 months, and cultured to observe the microbiological quality of the water. All the results (heterotrophic plate count at 22 °C, at 37 °C, and specific pathogenic germs sought) showed an absence of contamination. The protocol presented was effective over time and allowed ensuring the safety of care to be ensured when using MDDS, even during dental procedures under GA. As a result, it could be implemented by any dental care delivery structure wanting to reinforce the safety of its practice.

## 1. Introduction

**Figure 1:**
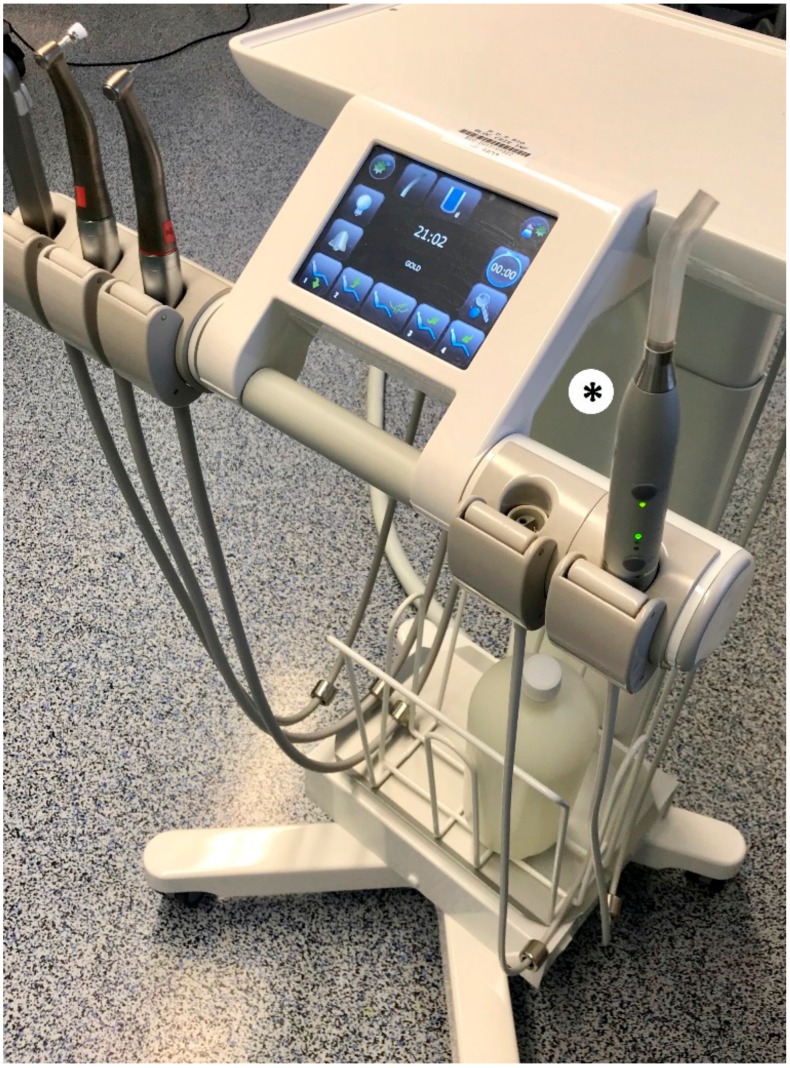
Mobile dental delivery service. * water reservoir.

Even if most dental procedures are performed in private practices, mobile dental delivery systems (MDDSs) (Figure 1) are receiving growing interest in the field of dentistry [1,2]. Many reasons can be attributed to this interest. Indeed, MDDSs associated or not to mobile units or vans allow unserved populations to be reached [2], providing cost-efficient services even if the time spent in setting up the unit could be a limitation [3]. MDDSs enable some barriers to accessibility to be broken, such as geographical barriers: patients from rural communities, isolated areas, etc. [1,2,3,4]; socio-economic barriers: children and elderly from families with low socio-economic status [1], patients medically underserved in poor urban areas [1], immigrants [1,5], homeless people [1]; and health barriers: vulnerable patients at home or in care facilities [1], hospitalized patients [1,3], and elderly patients [1,3]. In addition, to reach patients who live in areas not provided with dental surgeons, or to gain access to destitute and vulnerable patients, MDDSs can be used in a hospital environment, from room to room, or associated to the delivery of dental care under general anesthesia (GA) in an operating room. 

Many patients require dental treatment benefitting from GA [6], mostly patients feeling fear or extreme anxiety [7,8,9], with an excessive treatment need [7], or with an extremely severe dental problem [8,10]. Some patients accumulate several indications for GA dental procedures, especially uncooperative children [7,8,11]; physically and/or intellectually impaired patients with increased rate of tooth destruction due to trauma, tooth grinding, or acid regurgitation [9,12]; or patients with ineffective conscious sedation [7,13].

GA is a comfortable procedure for both the patient and the dentist, avoiding any cooperation problems, taking only a single appointment [7], and having a positive impact on parental emotion and conflict in case of young patients [8], but it is not without risks. Indeed, pain, nausea, and dizziness represent the major morbidity items after dental procedures under GA [9,14]. Death is also a possible complication, even if it is rather unlikely to happen [14]: during the years 1999 to 2005, there was a mean of 34 deaths per year due to GA among all the children who benefitted from GA in the USA [15]. However, there is a growing demand from patients to be treated under GA, leading to a long wait of several months, which increases the consumption of painkillers or antibiotics and the complications for patients [16]. In fact, several studies have focused on these delays: in the Netherlands, Boehmer et al. (2003) highlighted that the demand for GA exceeds the supply and that the waiting time was on average 8 weeks [17]. In the United Kingdom, according to Albadri et al. (2006) [18] and Lewis et al. (2002) [19] the waiting time was 10 weeks on average. For Foster Page in New Zealand, the average time was 2.8 months or approximately 11.5 weeks [20] between 2001 and 2005. For Haubek et al. in Denmark, the average time is 16 weeks [21]. In Australia, waiting times of 8 to 12 weeks are described, and in the USA, this time is 10 weeks on average [19].

Thus, dental procedures under GA, often associated with the use of an MDDS, represent a public health matter. Because concerned people are mostly vulnerable and weakened, and because there are comorbidities associated with these procedures [9,22], it is important that dental treatment under GA is performed as safely as possible [14]. For example, some cancer patients or hemophiliac patients could require hospitalization and could undergo dental procedures under GA. Based on the high risk of bleeding in patients with coagulopathies, guidelines have been established, particularly including the use of antibacterial mouthwash in the preoperative part, but no mouth rinsing for 24 h in the postoperative period [23]. As such, ensuring the diminution of the bacterial load in these vulnerable patients’ mouths is important.

This point leads to a focus on the hygiene of equipment such as MDDSs, in order to reinforce the safety of care. An MDDS is composed of the dental unit itself, a suction system, external surfaces, and dental unit waterlines (DUWL). If external surfaces and the suction system do not represent a challenge in terms of hygiene and disinfection when using appropriate products and methods [24,25,26], DUWL are regularly mentioned as the Achilles heel of the dental unit, due to its microbial contamination [27,28,29,30]. In fact, many studies report the presence of planktonic or sessile bacterial flora in DUWL, coming either from the water supply network [27,29], or as a result of a back-contamination of DUWL, caused by a backflow of oral fluids when rotary instruments stop [28,30,31]. DUWL, which are long and thin pipes, do present an important surface-to-volume ratio. This factor, associated with the presence of a laminar water flow, can promote biofilm formation [32]. Consequently, there is a real risk of infection for the patient and the dental team, which can even lead to the death of weakened patients [27]. Moreover, regarding the waiting time of several months before an appointment for dental procedures under GA, it is inconvenient to have to stop using an MDDS because it is contaminated. This would lead to an even longer waiting time, and associated complications for the patients.

If the dental chair, in its rudimentary form, has been used since the 19th century [33], it is only in the second half of the 20th century that the contemporary dental units took shape. Their first innovations were only technical (power, ergonomics, choice of instruments, etc.), but hygiene improvements were achieved from the 1970s, especially regarding external surfaces and the suction system [26,34]. It is only during the 2000s that hygienists and dentists focused on the microbial quality of the water in the dental unit waterlines, but mostly for fixed dental units. Indeed, MDDSs were then scarcely used, but their utilization has been developed since.

**Figure 2:**
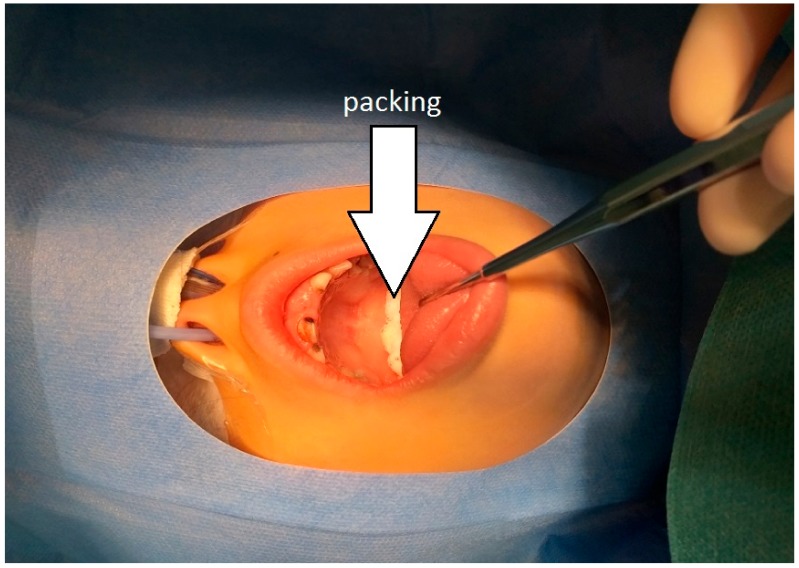
Pharyngeal packing in a child under general anesthesia (GA).

Once a week, dental practitioners from the Pediatric Dentistry department of the Strasbourg University Hospitals provide a session of care dedicated to children under GA, using an MDDS. Since the MDDS is used only one day a week, there is an increased risk linked to the possibility of backwater in the DUWL, and thus the development of a biofilm standing for a secondary reservoir of contamination. The use of a continuous water disinfection system previously showed good results in terms of microbiological quality control of the water that is used for dental procedures [30]. However, these results apply in general procedures, and there are no data about a prolonged contact of the disinfection product with the oropharyngeal mucosa during GA, especially regarding the possibility of its absorption in the presence of a pharyngeal packing (Figure 2). The latter is usually placed in the pharynx before starting dental procedures and is aimed to protect the airway from blood and debris, which could lead to obstruction and complications [35]. Therefore, we decided to use sterile water during these interventions and to implement a maintenance protocol to guarantee the safety of care when using MDDSs. Furthermore, even when using sterile water, a risk of water contamination in the DUWL still exists because of the possible back-contamination [28,30,31].

Thus, this study aimed to elaborate and assess over time this MDDS maintenance protocol, containing the management of dental unit waterlines and adapted to specific conditions such as dental care under GA.

## 2. Material and Methods

In the Strasbourg University Hospital, a new MDDS equipped with reservoirs for the water supply (Figure 1) was provided in February 2018 and reserved solely for dental procedures in children under GA. It is used one half-day a week, which corresponds to a mean of three patients per session. To ensure the safety of care, and in order not to allow the disinfection product, Calbénium© (Airel-Quetin, Champigny-Sur-Marne, France), to be in contact with the packing (Figure 2), the following protocol was elaborated and applied from February 2018.

### 2.1. Maintenance Protocol 

**Figure 3:**
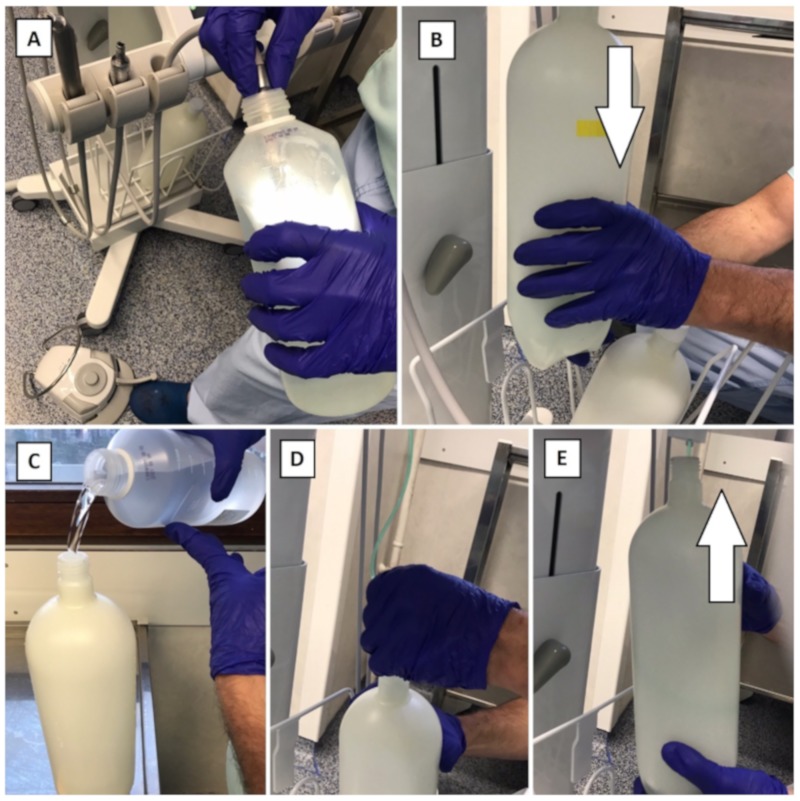
Parts of the maintenance protocol of the mobile dental delivery system (MDDS), before use and between each patient. (**A**) Purge of the air/water spray and the handpieces hoses with the Calbenium© solution; (**B**) removal of the reservoir that contains the Calbenium© solution; (**C**) filling of another reservoir with sterile water; (**D**,**E**) placement of the reservoir containing sterile water.

Before use:

- Remove the reservoir and disinfect it by soaking (as well as its cap) in a solution of Aniosyme X3© (Anios) for 5 min, in a tray dedicated to reservoirs.
- Rinse this reservoir and its cap with bacteriologically controlled water (tap provided with a bacteria filter), dry it with medical air, close it with its cap, and package it in a sterile pouch in order to stock it in clean and dry conditions.
- Fill another clean and disinfected reservoir with sterile water and Calbenium© at a concentration of 2%. Put it in place in the MDDS.
- Purge the air/water spray and the handpiece hoses for 1 min each in the suction can (Figure 3A).
- Leave for 5 min to act.
- During that time, remove the reservoir that contains the Calbenium© solution (which will not be used for the dental procedures), close it tight, and keep it for the next purges during the same session (Figure 3B).
- Put in place a reservoir, clean and disinfected, filled with sterile water (Figure 3C–E).
- Purge the air/water spray and the handpiece hoses in the suction can for 30 seconds each, to remove the Calbenium© from the DUWL.
- Clean and disinfect the surfaces using a detergent-disinfectant product.

Between each patient:

- Remove the reservoir and disinfect it by soaking (as well as its cap) in a solution of Aniosyme X3© during 5 min, in a tray dedicated to reservoirs.
- Rinse this reservoir and its cap with bacteriologically controlled water (tap provided with a bacteria filter), dry it with medical air, close it with its cap, and package it in a sterile pouch to stock it in clean and dry conditions.
- Put in place the reservoir that contains the 2% Calbenium© solution.
- Purge the air/water spray and the handpiece hoses in the suction can for 30 seconds each, before disconnecting and treating dental handpieces (Figure 3A).
- Leave for 5 min to act.
- During that time, remove the reservoir that contains the Calbenium© solution (Figure 3B), which will not be used for the dental procedures, close it tight, and keep it for the next purges during the same session.
- Put a reservoir in place, clean and disinfected, filled with sterile water (Figure 3C–E).
- Purge the air/water spray and the handpiece hoses in the suction can for 30 s each, in order to remove the Calbenium© from the DUWL.
- Clean and disinfect the surfaces using a detergent-disinfectant product.

After the last patient:

- Remove the reservoir and disinfect it by soaking (as well as its cap) in a solution of Aniosyme X3© for 5 min, in a tray dedicated to reservoirs.
- Rinse this reservoir and its cap with bacteriologically controlled water (tap provided with a bacteria filter), dry it with medical air, close it with its cap, and package it in a sterile pouch in order to stock it in clean and dry conditions.
- Put in place the reservoir that contains the 2% Calbenium© solution.
- Purge the air/water spray and the handpiece hoses in the suction can for 30 s each, before disconnecting and treating dental handpieces (Figure 3A).
- Leave for 5 min to act.
- Remove the reservoir that contains the Calbenium© solution (Figure 3B) and disinfect it by soaking (as well as its cap) in a solution of Aniosyme X3© for 5 min in a tray dedicated to reservoirs.
- Rinse this reservoir and its cap with bacteriologically controlled water (tap provided with a bacteria filter), dry it with medical air, close it with its cap, and package it in a sterile pouch in order to stock it in clean and dry conditions.
- Put a reservoir in place, clean, disinfected, and empty.
- Drain the DUWL with air for 30 s. Leave the reservoir in place.
- Clean and disinfect the surfaces using a detergent and a disinfectant.

The Calbenium© solution is composed of EDTA, benzalkonium chloride, sodium tosylchloramide, allantoin, aspartame, sorbitol, and spearmint flavor. Proportions of these products are not disclosed by the manufacturer. It is active against bacteria, fungi, and viruses according to standards AFNOR NFT72150, AFNOR NFT72151, AFNOR NFT72170, AFNOR NFT72200, and AFNOR NFT72180. 

Aniosyme X3© is composed of quaternary ammonium propionate, digluconate chlorhexidine, nonionic surfactants, and an enzymatic complex (protease, lipase, and amylase). It is active against bacteria, viruses, and yeasts according to standards EN1040, EN13727, EN14561, EN1275, EN13624, EN14562, and EN14476.

### 2.2. Study Design

Samples of the output water of the MDDS were collected at different times:

- J0, after a first realization of the maintenance protocol (“Between each patient” part), and before the first effective use of the MDDS in patients.
- J0 + 1 month, J0 + 3 months, J0 + 6 months, J0 + 12 months, and J0 + 24 months, to monitor the evolution of a potential contamination.

All these samples were collected in the morning, after the “Before use” part of the protocol, and before treating the first patient. Water samples culture conditions as well as the standards used [36,37,38,39,40] are described in Table 1. Microbiological quality levels were set according to European directives and hospital practice [36], and are even stricter than the standards given by the American Dental Association (ADA) [41]: compliant results with the expected values were viable aerobic microorganism count at 22 °C ≤ 100 cfu/mL, and at 37 °C ≤ 10 cfu/mL, and the absence of specific pathogenic germs (Table 1).

## 3. Results

Primary samples at J0, before any utilization of the MDDS with any patient, showed no contamination: <1 cfu/mL for the viable aerobic microorganism at 22 °C and at 37 °C, and no signs of, coliforms, *Escherichia coli* or *Pseudomonas aeruginosa*. The same microbial quality, <1 cfu/mL, was found for all the samples at J0 + 1 month, 3 months, 6 months, 12 months, and 24 months (Table 2), which means all these results were compliant with the expected values for the viable aerobic microorganisms at 22 °C and at 37 °C, the presence of coliforms, *E. coli*, or *P. aeruginosa*. Therefore, the protocol seems to be effective over time.

## 4. Discussion

Many cases of DUWL contamination are reported in the literature [27,30,40,42,43,44,45], and represent a concern for hygienists and dentists worldwide [45]. If previous studies showed good results of maintenance protocols applied to fixed dental units and dental chairs [29,44,46], the challenge was here to adapt a protocol to MDDSs for which water disinfectants are not used during dental procedures. Indeed, the MDDS was used under specific conditions such as GA environment and especially the presence of packing, and during procedures in vulnerable patients (children, impaired patients, etc.). In general, MDDSs are often used on-demand and not continuously: this causes less water flow and therefore an increased risk of forming a biofilm. If the constitution of a biofilm is never desirable, it is even less so for vulnerable patients.

In fact, the oral microbiota is complex, and a slight change can create an imbalance in favor of pathogenic species at the origin of some oral pathologies [47], especially for vulnerable populations or patients with low immune defenses. Maintaining a low level of bacterial load in a patient’s mouth, particularly for vulnerable patients, is important. For this reason, dentists usually use antimicrobial mouthwash before and after dental procedures or use newly developed interesting formulations, such as chlorhexidine gels [48]. In addition, diminishing the bacterial contamination of the water that is used for dental procedures seems to be a logical adjuvant. 

The protocol that we described is the first one ever described in the literature regarding the maintenance of MDDSs. Our results are far better than the expected values (Table 2) and show that the protocol and the products that we used were effective over time. The maintenance protocol could therefore constitute a basis for any dental care delivery structure wanting to reinforce the safety of its practice. The major flaw is that the protocol may seem rather complex at first glance. In a real application, however, the steps are easily sequenced, and users quickly get used to this protocol, with an average completion time of less than five minutes. Moreover, the application of the protocol can begin immediately after the end of dental care, and during the transfer of the patient in the recovery room, allowing benefiting from hidden time. This completion time is completely compatible with dental care activity, and ultimately represents a small constraint compared to the guarantee of the safety of the care, especially with vulnerable patients. To lighten this protocol, it would be interesting to look at the effects of prolonged contact of disinfectants with the oropharyngeal mucosa in order to avoid the use of sterile water without disinfectant during procedures, and therefore avoid several steps of the protocol. This seems complicated to undertake for ethical reasons, and because this protocol and our results show that there is a practically feasible solution. Nevertheless, it could allow using water from the hospital water supply network, just like it is used in fixed dental units with good outcomes in terms of microbiological quality of the water in DUWL [24,29], and allow moving towards greater efficiency, as sterile water is more expensive than water from the supply network.

## 5. Conclusions

The use of MDDSs meets many indications. It shows a growing interest in the dental care of isolated patients, as well as the provision of dental care in hospitals, from room to room, or during care under GA. When used with these fragile patients, the interest in guaranteeing the safety of care is then redoubled. Moreover, regarding the waiting time of several months before benefitting from dental procedures under GA, it is of utmost importance not to have to stop using an MDDS because it is contaminated. Different methods arise to manage the contamination of DUWL: filtration, flushing, use of sodium perborate or H_2_O_2_, or use of dedicated disinfectant solutions. Some have shown good results, and some show limitations. This study, using a Calbenium© solution, shows that a complete and well-managed hygiene protocol makes it possible to maintain over time a very low or even non-existent level of bacteriological contamination in the components of an MDDS and in particular in its DUWL. This protocol could be implemented by any dental care delivery structure that would like to enhance the safety of its practice and would like to avoid having to stop its activity for hygienic reasons. As such, it improves the quality of care for patients in need of dental procedures under GA and meets public health issues by reducing waiting time for appointments. 

## Figures and Tables

**Table 1:** Culture conditions of the output water samples collected from the MDDS and standards used [36].

Microorganisms Sought	Volume Analyzed	Maximum Storage Duration before Analysis Recommended (h)	Maximum Storage Duration before Analysis Accepted (h)	Storage Temperature (°C) before Analysis	Samples Seeding Conditions	Standard
Viable aerobic MO at 22°C	1 mL	8	12	5 ± 3	72 h at 22 °C on agar PCA by inclusion	ISO 6222 [37]
viable aerobic MO at 37°C	1 mL	8	12	5 ± 3	48 h at 36 °C on agar PCA by inclusion	ISO 6222 [37]
Coliform bacteria and *Escherichia coli*	100 mL	8	18	5 ± 3	24 h at 36 °C on agar TTC by membrane filtration, 2nd inspection after 48 h	ISO 9308-1 [38]
*Pseudomonas aeruginosa*	100 mL	8	12	5 ± 3 or ambient (≤25)	48 h at 36 °C on cetrimide agar by membrane filtration	ISO 16266 [39]

Abbreviations: MO, microorganisms; PCA, plate count agar; TTC, tergitol = medium used for the search and count of coliform bacteria.

**Table 2:** Results of the output water samples collected from the MDDS at different times.

Microorganisms Sought	J0	J0 + 1 Month	J0 + 3 Months	J0 + 6 Months	J0 + 12 Months	J0 + 24 Months	Target Level
Viable aerobic MO at 22 °C	<1	<1	<1	<1	<1	<1	≤100 cfu/mL
Viable aerobic MO at 37 °C	<1	<1	<1	<1	<1	<1	≤10 cfu/mL
Coliform bacteria and *E. coli*	<1	<1	<1	<1	<1	<1	<1 cfu/100mL
*Pseudomonas aeruginosa*	<1	<1	<1	<1	<1	<1	<1 cfu/100mL
